# Selection, adaptation, inheritance and design in human culture: the view from the Price equation

**DOI:** 10.1098/rstb.2019.0358

**Published:** 2020-03-09

**Authors:** Daniel Nettle

**Affiliations:** Institute of Population Health Sciences, Newcastle University, Newcastle upon Tyne, NE2 4HH, UK

**Keywords:** cultural evolution, cognition, selection, Price equation

## Abstract

For decades, parts of the literature on human culture have been gripped by an analogy: culture changes in a way that is substantially isomorphic to genetic evolution. This leads to a number of sub-claims: that design-like properties in cultural traditions should be explained in a parallel way to the design-like features of organisms, namely with reference to selection; that culture is a system of inheritance; and that cultural evolutionary processes can produce adaptation in the genetic sense. The Price equation provides a minimal description of any evolutionary system, and a method for identifying the action of selection. As such, it helps clarify some of these claims about culture conceptually. Looking closely through the lens of the Price equation, the differences between genes and culture come into sharp relief. Culture is only a system of inheritance metaphorically, or as an idealization, and the idealization may lead us to overlook causally important features of how cultural influence works. Design-like properties in cultural systems may owe more to transmission biases than to cultural selection. Where culture enhances genetic fitness, it is ambiguous whether what is doing the work is cultural transmission, or just the genetically evolved properties of the mind. I conclude that there are costs to trying to press culture into a template based on Darwinian evolution, even if one broadens the definition of ‘Darwinian’.

This article is part of the theme issue ‘Fifty years of the Price equation’.

## 1. Introduction: the culture debates

Some aspects of human behaviour are not direct consequences of genotype, and yet their properties seem to require appeal to something more than just idiosyncratic learning. For example, a person of Japanese descent growing up in California acquires Californian English, while her cousin in Japan acquires Japanese. We can obviously dismiss genetics as the cause of the difference in outcome. We correctly invoke learning instead. However, both Californian English and Japanese have super-individual, lineage-like properties not shared by other cases of learning. They have recurrent features that span many people and several lifetimes; there is both a chain of inter-personal continuity, and gradual change over time. Given this combination of super-generational continuity and gradual change, it is unsurprising that scholars have often turned to Darwinian evolution for paradigmatic metaphors. Culture appears to show Darwinian properties: something is inherited; something varies; and then there is differential proliferation and survival. The result is a changing population distribution of cultural items over time. Inspired by this isomorphism, Darwinian evolutionary models of culture were developed in earnest in the late twentieth century [1–4]. Though these models vary in how culture was conceptualized—in particular, how tight a similarity between the genetic and cultural cases is prescribed—they share enough in common to refer to them, henceforth, as cultural evolutionary theory.

A number of statements are made recurrently in summarizing the cultural evolutionary theory. One is that culture is a system of inheritance. Thus, humans have not just the standard one system of inheritance (genetics), but (at least) a second one: culture [5]. We have, in other words, a dual inheritance [3], and two inheritance systems entails two distinct fitnesses: genetic fitness and cultural fitness. Another statement is that cultural evolution produces design-like properties that would not emerge without it [6,7]. The key insight of Darwinian genetic evolutionary theory was that design-like properties could be produced, over time, by selection processes. Thus, it is quite natural, seeing design-like properties in culture, to assume they must be produced by selection processes too. Still another generalization is that cultural evolution can increase genetic fitness. For example, this claim is implicit in the idea that having a second inheritance system is adaptive for coping with environmental fluctuations faster than those that can be tracked by genetic selection, but slower than those generally tracked by individual learning (see e.g. [8]). ‘Adaptive’ in this context means genetically adaptive—more survival, more babies—and so for the claim to work, cultural evolution would have not only to increase cultural fitness, but genetic fitness too.

Cultural evolutionary ideas have been very influential, so much so that they are cited as a paradigm of what successful theory-building looks like [9], or drawn on in pursuit of other explanatory targets [10]. Yet at the same time, all of the main conceptual moves involved in likening cultural evolution to genetic evolution have always been [11], and continue to be [12–20], vigorously questioned. Some authors have sought to hang on to isomorphism in the face of the challenges. For example, they argue that the problematic features can be accommodated, since cultural evolutionary theory always admitted of differences between the cultural and genetic cases [21]. Others have argued that even though the analogy breaks down at the micro-evolutionary level (coming to hold an idea is not much like inheriting the short allele of the SL6CA4 gene), the parallel might work reasonably well at more macroscopic scales, where the details of the micro-mechanisms of transmission drop out of central relevance [19]. Still others have tried to retain the claim that cultural evolution is Darwinian by broadening the scope of the term ‘Darwinian’ [12].

This paper does not presume to adjudicate between competing claims about what if anything culture is, or how it should be treated in our accounts of human behaviour. What it will try to do is clarify some of the conceptual questions involved: what would have to be true for culture to constitute a system of inheritance; for design-like features of culture to be explained by selection; for cultural evolution to increase genetic fitness; and for cultural evolution to be Darwinian? A useful lens for conceptual clarification is the Price equation, which is what links this contribution to the others in this issue. The Price equation can be stated in various forms, with varying degrees of abstraction (see [22]). In its most abstract form, it offers a minimal description of an evolutionary system, and a way identifying how much of the change within it is owing to selection. Price predicted that a general mathematics of selection, to which his equation was an initial offering, would be equally applicable to genetic and to other kinds of system [23]. This leads naturally to its application to culture [24]. I will use a toy example of the cultural evolution of song, but the reader may substitute any other cultural example they prefer (the example is loosely motivated by empirical research in the cultural evolution of music [25,26]). In §2, I explain what the Price equation looks like for our cultural example. In §3, I turn to some contentious issues that the Price equation helps clarify: when directional change is owing to selection (§3.1); when and why culture could increase genetic fitness (§3.2); and whether culture is a system of inheritance (§3.3). Section 4 returns, in the light of §§2 and 3, to whether cultural evolution is best thought of as a Darwinian process.

## 2. The Price equation: a cultural application

If the Price equation is just an equivalence, or tautology, then why am I so enthusiastic about it?

[22, p. 1017].

**Figure 1.**
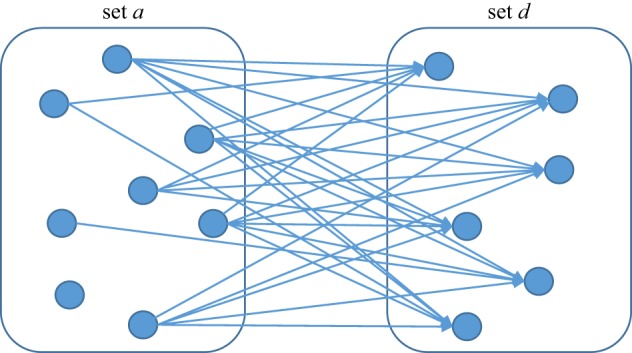
Schematic of the conditions required to apply the Price equation. There are two sets of individuals, *a* and *d*, and some trait measurable on each individual. There are directed links from some individuals in set *a* to those in set *d*, which represent influence on the value of the trait. Fitness is defined, for individuals in set *a*, as the number of outgoing links. The trait values of the individuals in set *d* may or may not be equal to the average of the individuals they receive links from. (Online version in colour.)

To apply the Price equation, there need to be two sets of individuals: the first set (*a*) forms the ancestral generation, and the second set (*d*), the descendant generation (figure 1). Each member of sets *a* and *d* must have a particular value of the trait for which the equation is to be constructed, in our case song style. Let us assume that every individual sings, and that their individual song style can be placed on a continuum describing the *entropy* of that song style. In a high-entropy song style, many different pitch transitions occur and there are many different note durations, and phrases do not repeat. It is thus very hard to predict either the pitch or duration of the next note from the context. In a low-entropy song style, a smaller set of pitch transitions and note durations is used, and whole phrases repeat. Hence, low-entropy songs are much easier to sing and learn. Low-entropy songs appear *well designed* for humans to acquire. We denote the entropy of the *i*th individual's song style *z_i_*.

There must be directed links from some individuals in the *a*-set to some in the *d-*set (figure 1). Links represent influences in song entropy going from the individual on the upstream end of the link to the individual on the receiving end, owing to emulation or learning. No genetic connections need to be assumed between individuals in the two sets; there might or might not be any. To apply the Price equation in its deterministic form (see [27]), we need to possess full information about which ancestors have influenced which descendants and how strongly, and the trait values (the *z*) for all of the *a*-set and all of the *d*-set. Note, therefore, that we can only apply the equation numerically once the ancestor–descendant influence has already happened and the phenotypes of the descendants are set. We are not, in this exercise, predicting the future, nor inferring the change in a whole population from data on a sample. Rather, we are producing an exact description of the population change that has happened in a single generation. As an exegetical simplification, I will assume that every descendant has the same number of ancestors (say five), and that the ancestors are all equally influential on the descendant's trait value. Neither of these assumptions is necessary (see [24] for a fuller treatment), but they make the presentation easier.

With our information on ancestor–descendant links and trait values in hand, there are several things we can do. First, we can calculate the change in average song entropy between the *a*-set and *d*-set. Denoting arithmetic mean with an overbar, then in this one generation of evolutionary change, average song entropy has changed by $\Delta \bar{z}={\bar{z}}_{d}-\;{\bar{z}}_{a}$. We can also calculate the (cultural) *fitness* of each individual in the *a*-set: this is the number of individuals that particular ancestor influences in the *d*-set. Therefore, we just count up the number of outgoing links from the *i*th individual to get that individual's fitness *w_i_*. Fitness is not defined for individuals in the *d*-set: that would require another set who learned from *d* in their turn. Having calculated the fitness of each *a*-individual, we could ask whether there is any relationship between their song entropy and the fitness they ended up achieving. Such an association is captured by the covariance between ancestors' song entropy and their fitnesses, cov(w,z). A covariance is just an unstandardized correlation, hence positive if those with higher-entropy songs had higher fitness, negative if those with higher-entropy songs had lower fitness and zero if there was no relationship at all between song entropy and fitness. The existence of a non-zero covariance between song entropy and fitness indicates that there is *selection* on song entropy. It is not just a correlate of selection; it is what it means for there to be selection [28].

A final thing we might wish to calculate is how the song entropy of descendants relates to the song entropy of their ancestors. There are several possibilities here. Each individual in the *d*-set might have the average of the song entropies of the individuals in the *a*-set that influenced them. If this were the case, or even if there were some random noise, then, representing the difference in song entropy between ancestors and their descendants as Δz, E(Δz)=0. On the other hand, in the process of transmission, the trait values of the ancestors might get transformed. When singing songs they have learned, individuals might sometimes forget a few details of what they heard, substituting a slightly more predictable pitch or duration (or even just repeating a whole phrase if they could not remember the next one). In this case, then the songs they produce would, on average, have slightly lower entropy than the ones to which they were exposed, and hence E(Δz)<0. (One could also imagine scenarios where E(Δz)>0, where each singer elaborates on learned song forms to impress their friends. The direction of the transformation is unimportant for the conceptual point.) E(Δz) is readily calculable with the information we have already discussed.

One version of the Price equation for this scenario is

2.1 $$\bar{w}\Delta \bar{z}=\text{cov}(w,z)+E_{w}(\Delta z)$$

Here, $\bar{w}$ is the average fitness of ancestors across the whole population (effectively this is just a normalizing constant, 5 in this case) and $E_{w}$ represents a fitness-weighted expectation, rather than the simple expectation. On the left-hand side, we have the change in song entropy from the *a*-set to the *d*-set $\left(\Delta \bar{z}\right)$. On the right-hand side, we have two terms: the covariance between song entropy and fitness (the selection term) and the expected value of the difference in song entropy between a person in the *d-*set and their influencers in the *a-*set (the average transmission term).

What have we achieved at this point? Both the left- and right-hand sides of (2.1) consist of things we were able to calculate from the information we already possessed. The equation thus does not estimate any currently unknown parameters, still less make any predictions about the change in song entropy likely to occur in the next cultural generation, for whom the covariances and expectations might be different (this is what is often referred to as the ‘dynamical insufficiency’ problem of the Price equation [22]). Thus, the right-hand side simply represents the same information as the left-hand side in a different format. This deflationary view is what motivates the critique that the Price equation is trivial or useless in practice [29].

This critique, however, misses the point. The Price equation is, indeed, a mathematical tautology [22]. It does not *predict* the change in song entropy from one generation to the next. Instead, it merely shows that the change in song entropy *can always be rewritten* as the sum of a covariance and an expectation. This rewriting, while not producing any new information, can be epistemically useful. On the left-hand side, we can see whether there has been any evolutionary change from one generation to the next in this particular population. Until we perform the rewriting of the right-hand side, we cannot see whether that change is owing to selection, or to something else. Equation (2.1), by providing a general decomposition of the sources of cultural evolutionary change, can help us clarify some of the issues raised in §1. The next section discusses how it does this.

## 3. Conceptual issues in cultural evolution from the perspective of the Price equation

### (a) Are the design-like features in culture produced by selection?

Imagine we make the observation that, in each successive cultural generation of singers, the average entropy of songs becomes lower. This goes on over many decades, to the point where the distribution of song entropies is much too concentrated at the low end to be owing to chance. This is the emergence, through evolution, of a design-like property: low-entropy songs are easier for humans to retain and sing. In genetic evolution, observing the gradual emergence of design-like properties such as a streamlined body shape in an aquatic animal, researchers' first intuition is to reach for selection as the relevant explanatory construct. Cultural researchers, understandably, are tempted by similar moves. If the population becomes increasingly dominated by low-entropy songs, then it seems like there must have been cultural selection for low entropy over the generations (an assumption embodied, for example, by MacCallum *et al*. [26]). Darwin himself was tempted by such a move, claiming in a widely cited passage in *The Descent of Man* (p. 90) that the emergence of certain word forms in language change must be owing to selection [30].

However, the appeal to selection is only sound for the genetic evolutionary case because, there, the selection is the only plausible source of the systematic change. In genetic biology, faithful DNA replication, fair meiosis and the randomness of mutation with respect to function ensure that, except for special cases, the average transmission term on the right-hand side of the Price equation is zero; it drops out. What we are left with is directional change in phenotype implying selection, and selection implying directional change in phenotype.

In the cultural case, however, we cannot so easily write off the average transmission term. Humans have learning biases, attentional limitations, non-zero priors, memorial foibles, deliberate strategies and so on. Thus, in general, people will produce things that are not just different from the sum of things they learned, but different in consistent directions. Change through average transmission is almost guaranteed to be more important in the cultural than the genetic case, for two related reasons. First, the proper function of DNA replication mechanisms is to replicate, and they do so indifferently to the content of the message they are replicating (they are, for example, indifferent between different nucleotide bases, and to whether the sequence they are replicating will ever be transcribed). The same cannot be said of humans: they replicate culture, if they do so at all, usually in the course of achieving a wide range of other purposes, other purposes that usually prescribe or favour transformation [20]. Second, replication is, in the genetic case, direct: an actual physical copy of a molecule is made. In the case of cultural contents, replication or transmission are only metaphors for processes that are actually indirect [18]. Someone in set *a* produces an action or artefact in the public sphere. Another person in set *d* perceives and appraises this, making use of inferential and other cognitive abilities, and this affects their internal cognitive states. They may then later produce an act or artefact of their own, guided by those cognitive states and hence indirectly by the prior action or artefact. This action or artefact is not, under any circumstances, a physical copy of the earlier act or artefact. Inferential and other cognitive mechanisms have intervened. DNA replication intervenes in genetic transmission, of course, but the effects of its intervention can be captured for many purposes with a very thin description: basically, it just replicates the molecule. The parallel thin description for human agents—basically, they just copy—is less likely ever to be adequate.

**Figure 2.**
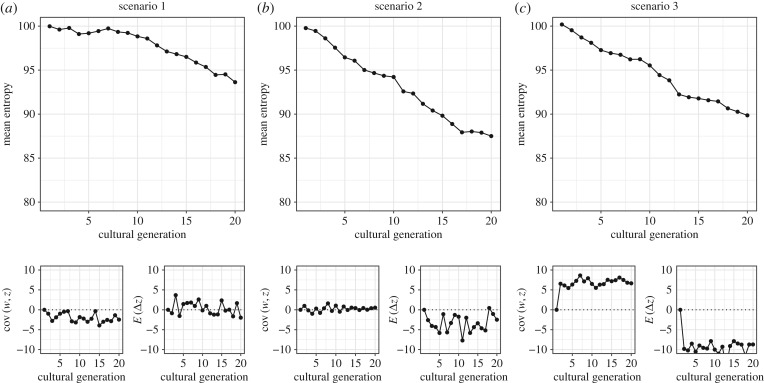
Simulations of change in the entropy of song style under three evolutionary scenarios. In each column (*a*–*c*), the main plot shows the change in the population mean of the entropy of song styles over 20 generations. The bottom left inset shows the covariance between fitness (number of learners each ancestor attracts) and entropy. The bottom right shows the mean value of the entropy difference between a descendant's productions and the average of those of their ancestors. Panels (*a–c*) correspond, respectively, to the three scenarios explained in §3a. All simulations are based on a population size of 1000, with each descendant learning from five ancestors.

Once we have non-zero average transmission, the Price equation tells us that selection alone does not determine the rate or even the direction of evolutionary change. To make this point, figure 2 shows simulated populations of 1000 singers over 20 non-overlapping cultural generations, under three evolutionary scenarios (see electronic supplementary material for simulation methods and code). In all scenarios, each singer in the new generation samples five singers to learn from in the previous generation. In the first scenario (column (*a*) of figure 2), sampling is related to the entropy of that individual's song: those with lower-entropy songs attract more learners. For this reason, there is a consistently negative covariance between song entropy and fitness (lower left panel). The scenario also assumes that the entropy of a learner's song is on average just the same as those from whom they learned (hence an average transmission term around zero, lower right). The effect is that the entropy of song systems reduces over the cultural generations (main panel of column (*a*)). This reduction is owing to cultural selection, exactly because the covariance term of the Price equation is consistently negative.

In the second scenario (column (*b*)), learners choose who to learn from entirely at random, with no consistent relation to their teachers' entropy. Hence, the covariance terms tend to hover around zero. However, through their memorial lapses and spontaneous regularizations, learners tend to produce songs with entropy that is just slightly lower than their models; hence the consistently negative average transmission terms. The consequence is a sustained reduction, over the generations, in song entropy.

Finally, in the third scenario (column (*c*)), there are two forces. Singers whose song entropy is *high* attain virtuosic prestige. Their song styles are considered refined owing to their sophistication. More prestigious singers recruit more learners, generating a positive covariance between entropy and fitness. However, learners do not manage to perfectly reproduce the entropy of their virtuosic teachers; they forget some of the surprising transitions and changes in pattern, filling in with something more obvious, and thus producing songs lower in entropy than those they were trying to emulate. What happens over the generations is a sustained reduction in song entropy, despite selection for it to increase. The covariances (bottom left for each figure part in figure 2) are consistently positive, but they are coupled with even larger negative expectation terms (bottom right). Since the Price equation is just a sum, the term with the larger absolute magnitude is the one that wins out.

The moral of this story is that, in the cultural case, where we observe directional change towards traits that appear well-designed for human use, we cannot infer that cultural selection is responsible. The three main plots of figure 2 look almost identical, but in the first, the selection is the explanation; in the second, there is no selection; and in the third, there is ongoing selection in the opposite direction. This makes problematic the argument that we can adapt the population-level ‘tests’ used to identify genetic selection in natural populations to infer the operation of selection processes in the cultural domain (see [7] for a discussion). Since average transmission terms in cultural evolution are likely to be non-zero, the mere observation of apparent design, or directional change over time, is inconclusive as to the force producing it. Likewise, we cannot assume that cultural evolutionary processes maximize cultural fitness, whereas the parallel assumption for genetic evolution is generally safer [24]. The operation of selection could only be identified, in the cultural domain, if cultural fitness and hence its covariance with trait values could actually be measured. This is not straightforward, since identifying and quantifying cultural influence are not nearly as simple as counting genetic offspring, which evolutionary biologists do routinely (see §4 and [12,24]).

The fact that non-random cultural change might represent transmission or selection, or any combination of the two, has been well made before [31]. However, the Price equation, with its separation of change into two additive terms on the right-hand side, makes it particularly clear what the driving forces are. In historical case studies (e.g. [32]), it is easier to detect directional cultural change than it is to definitely assign it to either selection or transmission. There are only a few cases where researchers have designed paradigms that isolate one force. For example, one can compare MacCallum's [26] public music choice experiment, which allowed selection among computer-generated tunes but no average transmission bias, with Ravignani and Delgado's transmission experiment, which allowed bias in transmission but no selection [25]. In both studies, sustained non-random change was observed, in the direction of features that recur in human musical traditions. Eriksson and Coultas [33] investigated the directional emergence of disgusting content in transmitted stories. They isolated average transmission (the difference between the story a participant received and the version they subsequently produced) and selection (which of several stories a participant chose to read), and showed that both were biased toward the stories with higher-disgust content. (Confusingly, the authors refer to these two forces as two phases of cultural selection, though one is clearly average transmission rather than selection.)

These examples suggest that design in cultural evolution is produced by both transmission and selection. Their importance may not be equal, however. Experimental work using iterated learning, or transmission chain designs suggests a very large role for average transmission. In these experiments, one participant receives a stimulus (for example, a story), and after a short delay reproduces it. The next participant receives the first participant's output, and so on in chain-like fashion. There can be no selection in iterated learning experiments: the fitness of every ancestor is identical (one descendant). The results show, first, that transformation effects are very large. People do not faithfully replicate; so much so that within a few generations, the resulting product often bears little relation to its founding ancestor [34]. Second, transformation effects occur not at random, but in systematic directions; so there is evolution in the absence of selection. Third, most importantly, non-obvious design-like features of human cultural systems emerge in these experiments, quite quickly and without apparently being strongly seeded by the experimenters. Examples include aspects of language structure such as compositionality, grammatical regularity and animacy distinctions [35–37]; conceptual categorization imposed on underlying continua [38]; the rhythmic universals observed in music [25], verse-metre conventions [39] and simplifications of cause–effect relations [34].

If we accept the validity of these laboratory scenarios as models of naturally occurring cultural processes, the findings pose a challenge. If it is possible to generate so many of culture's non-random properties without allowing for any selection, then how important is selection for explaining patterns of culture? If the transmission is doing most of the work, then the rhetorical grip of the genes–culture analogy is loosened; cultural evolution is just like genetic evolution except that the design-like properties mostly arise for a different reason. I return to this issue in §4.

### (b) When does culture produce genetic adaptation?

There is a Manichaean tendency within the cultural evolution literature. On the one hand, it is clearly understood that cultural fitness is to do with one's ability to influence the cultural contents of minds in the future generation, and as such has nothing necessarily to do with number of biological offspring. On the other hand, it is often asserted that culture is an adaptive capacity, and that through it humans have been able to survive and proliferate better than they otherwise would. This implies that researchers believe cultural evolutionary processes can have an impact on genetic fitness. The Price equation is useful for specifying under what conditions this will happen.

A simple way of doing this is to construct a trait *g* that describes ‘doing all the things that, in this environment, lead to high genetic reproductive success'. What will be the impact of cultural evolutionary processes on the population mean of *g*? From equation (2.1), we have

3.1 $$\bar{w}\Delta \bar{g}=\text{cov}(w,g)+E_{w}(\Delta g)$$

A covariance can be rewritten as the product of a variance and a regression coefficient, giving

3.2 $$\bar{w}\Delta \bar{g}=\beta (w,g)\text{var}(g)+E_{w}(\Delta g)$$

Here, β(w,g) is the regression coefficient of genetic-fitness-maximizing-behaviour on cultural fitness; effectively the correlation between social influence and lifetime reproductive success. If this coefficient is positive, then (in the absence of a countervailing transmission term) cultural selection will lead to the behaviours that produce higher genetic fitness becoming more widespread. Population mean genetic fitness will increase. A positive coefficient could arise through biases to emulate just those individuals who are successful in some genetically relevant way, like surviving or attaining status or resources [3]. However, negative coefficients could also occur. If there is a strong trade-off between becoming socially prestigious and having children, as may be the case for example for career success in modern industrial societies, then β(w,g) becomes negative and cultural selection will reduce genetic mean fitness. This is a candidate explanation for the historical emergence of small family sizes (see [3, pp. 199–221]).

The Price equation lets us see that there is also another pathway for cultural evolution to increase genetic fitness. Biased average transmission (the second term on the right-hand side of (3.2)) can also produce directional changes in genetic fitness. If humans have predispositions or priors concerning certain behaviours, because those behaviours have usually been genetically fitness-enhancing over evolutionary time, or just the ability to learn from consequences, then the effect will be to shift average cultural practices in the direction that increases genetic fitness. Biased average transmission, in effect, provides a universal force of attraction towards practices of the kinds that work adaptively over evolutionary time, or at least, a source of resistance against cultural practices that are too outlandish from the perspective of past genetic fitness. Biased average transmission means, in effect, that although patterns of culture end up containing practices that are good for genetic fitness, it is not cultural evolution that is doing the fitness-enhancing work; it is prior *genetic* selection on the cognitive mechanisms that transform culture. Culture effectively drops out of the equation, an intermediate variable between the adaptive behaviours and their true organizational source [40]. Much of the debate between cultural evolutionary theory on the one hand and evolutionary psychology on the other, through the window of the Price equation, is a debate about whether the first or second term on the right-hand side of (3.2) is more important in explaining patterned human behaviour.

### (c) Can culture be said to be a system of inheritance?

The Price equation is usually thought of as defining the conditions for selection to be an evolutionary force. However, it also offers conditions for being able to describe a system as involving inheritance. In figure 1, there is inheritance for two reasons. First, the links from *a* to *d* are directed; there are no links back from *d* to *a.* Second, it is possible to index which members of *d* were influenced by *a*, and how strongly. Without these two conditions being met, it is hard to think of the system as involving inheritance and, relatedly, the whole notion of fitness ceases to be well defined—in the terms of [19], there would be no *Darwinian individuals*.

It is not clear that figure 1 captures cultural processes between people. Person A suggests an idea for a theory; person B thinks about this idea, writes a draft paper and sends it back to A. A reads it and realizes that the theory needs revision. A tells B this in an email and B sees A's point, changing her presentation of the theory in a subsequent talk. Though there is clearly social influence in this scenario, there is no inheritance, exactly because the link is bi-directional. Hence, there is no simple measure of the cultural fitness of an individual. A and B influenced one another repeatedly in the course of coming to hold certain cognitive representations. (‘Horizontal transmission’, a building block of cultural evolutionary theory, does not describe this situation, but rather, unidirectional influence from an ancestor to a descendant who is not a genetic relative.)

The issue is obvious once pointed out. So how does cultural evolutionary theory deal with it? Classic dual-inheritance models [3] make the idealization that cultural traits are only transmitted once in the human lifetime. Cognitive transformation is applied to whatever content is acquired (this is referred to as ‘guided variation’). However, this happens only once the transmitted material is safely inside the receiving mind. The process of transmission between minds happens just once for all time, with no negotiation and no back-flow. This idealization is what makes the concept of inheritance, and much of the machinery of population genetic modelling, applicable.

There may well be cultural cases for which the idealization is adequate. The phonemic contrasts of a native language are acquired early in life and may not be much changed thereafter. Moreover, they are acquired without any back-and-forth reasoning or debate. Thus, describing the phonemic contrast system of a native language as something that is inherited might be reasonable: before you are a fully competent speaker, you are in descendant mode; and once you have learned, you become available as a potential ancestor. It is not clear that the idealization is adequate more generally, though. Skilled performance, moral judgement and political preferences can change dramatically over the course of an individual's lifetime, exactly because there is constant reshaping and discussion going on between multiple individuals, none of whom is wholly an ancestor or wholly a descendant. The transformative effects of human cognition—that which in equation (2.1) we tried to capture with the average transmission term—are in fact produced in multiple interactions across multiple minds. The problem with seeing cultural influence as inheritance is that the social part gets restricted to the once-in-a-lifetime, ancestor-to-descendant transmission. The cognitive part gets restricted to the guided variation individuals may apply within their own skulls. What is missing is the dialogic activity underlying the (constant, ongoing, inter-personally negotiated) transformation of cultural content. This dialogic part may matter for the most distinctive aspects of human culture and institutions. It may turn out to matter even in some cases that we usually think of as inheritance. In Creole languages, for example, the lexicon and phonology may be inherited in the sense outlined above. The morphology, however, emerges through repeated interaction between peers who have no inheritance to build on, but have a mutual desire to communicate [41].

Once the possibilities of repeated mutual influence and dialogue are admitted, simple notions like cultural fitness are no longer calculable. The cultural traits that become prevalent in the long term may not be those that attract the most learners initially [16]. One could think, for example, of populist political policies that are intuitively appealing on first hearing, but fall apart the moment they are subjected to sustained argumentation. Would they spread or not? It would depend on the ratio of reasoned conversations to sound-bites in a particular social network. Is the fitness associated with such ideas high or low? It depends whether you measure, as fitness, the initial attraction probability, or the resistance to abandonment following argumentation; both will have an impact on the equilibrium prevalence of the policies. In short, thinking of cultural representations as being acquired through one-off inheritance might preclude the study of important causal forces accounting for the structure and dynamics of cultural representations.

One move often made to salvage the idea of cultural influence as inheritance is to switch the individuals represented in figure 1. For example, the individuals in *d* could be renditions of a particular song, and the individuals in *a* could be earlier song renditions. A relationship of inheritance would hold from the earlier rendition to the later one influenced by it. Fitness would be the sum of influence on future renditions of a given earlier rendition. This works up to a point. Since later renditions can only be affected by earlier ones, there are no problems of bi-directionality. How useful this notion of inheritance turns out to be still depends on how the transmission works. If every rendition can be very clearly linked to a small number of earlier renditions and not others, then ancestry and descent are fairly clear. If, however, current renditions are somewhat influenced, in different ways, by a very large proportion of earlier ones within that social group, then the ancestor–descendant metaphor loses utility. As Godfrey-Smith puts it: if there are too many parents, there are no parents at all [19]. This is especially true where there is a large amount of active reshaping of the transmitted material, and becomes even more acute once we admit that song renditions can be influenced by a potentially limitless set of things that are not song renditions at all (e.g. films, world events, natural or industrial sounds). Measuring fitness, even at the cultural-item level, then becomes problematic.

Even if the move from people to song renditions as the Darwinian individuals proved fruitful, it would still be wrong to claim that humans have a second system of inheritance running alongside the genetic one, or a dual inheritance. In the rendition-as-individual idealization, humans are not inheritors of culture. They are just the ecological background, providing selection pressures on cultural renditions through their tastes and propensities. We can retain the notion that humans are the individuals whose phenotypes we are studying, but in this case, we must recognize that their acquisition of culture is not like the inheritance of their genes, and so they only have a dual inheritance as a metaphor or idealization. Alternatively, we can move to modelling a world where humans—one type of Darwinian individual evolving with one system of inheritance (genes)—are hosts and ecological backgrounds to the propagation of another type of Darwinian individual (song renditions), which also has one system of inheritance.

## 4. Is cultural change Darwinian?

The considerations in §3 bring us back to an overall assessment of whether cultural change is best thought of as a Darwinian process. Space precludes a full review of all the possible and actual answers (see [7,12,17,19–21]). These depend on what scope we give the term ‘Darwinian’: we can always define it in such a way as to include the key features of a cultural case we are interested in. Thus, the question is not whether we *can* say that cultural change is a Darwinian process, but how epistemically *useful* such a stance is.

Cultural evolutionists have always stressed that cultural transmission is not exactly like genetic transmission (see e.g. [1,3,7,42]). Indeed, much of their work consists in documenting the differences and suggesting how to model them. What unites them is the notion that *despite the differences*, a parallel with Darwin's account of genetic evolution is still a helpful starting point. Another view is that the differences are so fundamental that we would do better to begin our study of culture completely unencumbered by the analogy. As this view has been less often defended than the other, I will briefly summarize some arguments for it. We start by asking: what is Darwinian about Darwin's theory of genetic evolution?

For living things, there are unidirectional relationships of ancestry and descent, as shown in figure 1. The modes of ancestor–descendant relationships are stable and straightforward: for us, for example, all descendants have exactly two parents, weighted almost equally. For selection, and hence the Price equation, to apply there need to be clear Darwinian individuals with simple, unambiguous ancestor–descendant links: if there are too many parents or their influence is obscure, there are no parents at all. Let us call the presence of discrete individuals with unidirectional, countable ancestor–descendant links the *Darwinian pre-requisite*.

The Darwinian pre-requisite is necessary for Darwin's theory, but not sufficient to characterize it. Darwin worried about the problem of design, namely where the non-random functional characteristics of organisms had come from, but he was by no means alone in suggesting they emerged gradually through an evolutionary process. Others had done so too, but in different ways. Prototypically *non*-Darwinian accounts, like that of Lamarck, attributed the design-like properties to the average transmission term of the Price equation. Though Lamarck is most associated in contemporary discourse with the inheritance of acquired characteristics, there is a second important component of his theory, that of mutation to address a felt need. In other words, in Lamarckian evolution, directional changes stem from the average transmission term: organisms systematically move their type in a non-random direction. Darwin's defining insight was that this was not necessary. The average transmission term could be zero, and yet design-like properties still emerge, driven by the selection component. This is not to argue that Darwinian evolution admits of no forces other than selection. It is to argue that Darwin, distinctively, accords a central role to selection rather than transmission in explaining the emergence of design-like properties.

Viewed in this light, it would seem reasonable to reserve the category ‘Darwinian’ for cases where the Darwinian pre-requisite is satisfied *and* there is a prominent role for selection rather than transmission in explaining design-like properties. One need not, perhaps, go so far as to refuse the designation ‘Darwinian evolution’ to all cases where the average transmission term is non-zero. However, there is no doubt that the success of Darwinian evolution as an explanatory paradigm owes much to the fact that, in genetic cases, it has generally turned out to be zero. The greater the importance of average transmission in explaining design-like properties, the less the similarity to Darwin's theory becomes. One could loosen one's definition of ‘Darwinian’ to include such cases (for example, calling all populational processes over time ‘Darwinian’ [12]). However, any such broad notion of ‘Darwinian’ would also include accounts of the genetic evolutionary process that historians of science see as importantly *different* from Darwin's.

In this paper, I have drawn attention to key differences between genetic and cultural processes: in culture, much of the design work may be done by average transmission; ancestor to descendant relationships may not be straightforwardly verifiable matters; and ‘inheritance’ followed by ‘mutation’ is only a metaphor or idealization for a range of processes occurring within and between minds (see also [43] for further elaboration). This means that key concepts like fitness and selection could be hard to measure in practice, and possibly even undefined in theory. In view of this, there would seem to be a reasonable case for not saddling ourselves with the analogy to genetic evolution, but just making tools for the cultural case that are grounded in the natural properties of that case. This certainly does not mean that all the results of prior cultural evolutionary work are wrong. Any attempt to model culture as a populational phenomenon can lead to valid insights, even if based on idealizations that are eventually discarded. Moreover, as Godfrey-Smith points out [19], even if the genes–culture analogy fails at the micro-evolutionary level, it can lead to useful generalizations and methodological advances at more macroscopic levels of analysis, such as that of language phylogenies. Nonetheless, advances in understanding the population distribution of cultural practices or representations seem most likely to come from cognitive science: understanding in detail what kinds of cognitive representations people hold, what capacities and priors allow them to do this and how social experience updates these representations. Such understanding could build out from the properties of the individual knower to the trait distributions arising in interacting networks of individuals (see [44] for a promissory example). No isomorphism to Darwinian evolution is required in order to do this (see also [45]).

Perhaps it does not matter whether we adopt the genes–culture analogy or not. One researcher starts from the genes–culture analogy and progressively builds the differences between genes and culture into her models. Another starts from cognitive science with no Darwinian assumptions, and builds up to populational phenomena. They may converge on the same insights. However, foundational metaphors are important for a number of reasons. First, they are pedagogically influential. When we explain our theories, we start with ‘culture is like genes', and then add the nuances. People forget or fail to hear the nuances; they only remember the framing, which they then either contest or apply too literally. Thus, theories of culture whose first move is the analogy with genes may be condemned forever to spend most of their time re-explaining their spoken-but-not-heard nuances.

Second, foundational metaphors bias where we turn to, seducing us down some paths and leading us to overlook others. When we see design or directional change in culture, we too readily reach for selection, since that is what our foundational metaphor makes most cognitively available. Darwin did so, for word forms, and may well have been wrong. We do not have the right intuitions for the importance of transmission exactly because transmission is not what is important in the genetic case. Likewise, the processes whereby adult humans continually, mutually and reciprocally affect each others' behaviours and cognitive states, through mechanisms such as shared activity and argumentation, have been paid too little attention in the cultural evolution literature. This is because they do not fit with the simple concepts of inheritance or mutation (see also [20]). A central critique of cultural evolutionary theory, from both the anthropological and the cognitive science perspectives [45,46], is that the description of humans it assumes is too thin—they just imitate, and there is not much more you need to know, except that their imitation could be biased in a few simple ways. This follows directly from the foundational metaphor, since a thin description—they just replicate, plus a bit of mutation—works pretty well for the genetic case. With a different, more realistic foundational metaphor, perhaps we would move more quickly towards theories that attribute the individual humans with more of the thickness and complexity required for adequate accounts of human behaviour.

Others have made these points before, and yet the hold of the genes–culture analogy persists. Perhaps this is just because it is simpler and more transmissible than the alternatives. Darwin's theory of genetic evolution is a very successful theory, and people at least think they understand it (though in fact they often do not [47,48]). Critiques of its application to culture can seem tantamount to saying ‘it's all very complex and what happens depends on the details'. Hence, they are relatively unattractive. But the case underlying them seems strong: explaining culture involves all the problems of cognition (how do minds come to know and do the things they know and do?) shackled to social science's long-standing micro–macro problem (how do the properties of individuals affect those of their social groups, and *vice versa*?). This endeavour is not precisely analogous to any other.

## Supplementary Material

Description of simulation methods

## Data accessibility

Code for simulations in figure 2 is available at: https://osf.io/n695v/
