# Supplementary material for: Selection, adaptation, inheritance and design in human culture: the view from the Price equation
Source: Philos Trans R Soc Lond B Biol Sci. 2020 Mar 9;375(1797):20190358. doi: 10.1098/rstb.2019.0358 (PMC7133501; doi:10.1098/rstb.2019.0358)
Supplement: Description of simulation methods [file rstb20190358supp1.pdf]

Supporting information for 'Inheritance, selection, adaptation and design in human culture: The view through the Price equation window'

Daniel Nettle

This short supplement describes the simulations that produced figure 2.

General approach

First, I generated 1000 individuals with song entropies drawn from a normal distribution with (arbitrarily) mean 100 and standard deviation 10. These individuals form generation 1.

The song entropies of generation 2 are generated as follows. For each individual in the generation, I sampled 5 individuals from the previous generation to be the ancestors (on the selection of these, see next section). The average entropy of the songs of the ancestors was calculated. A transmission term was then added to this average. The transmission term was drawn from a normal distribution with mean as specified in next section, and standard deviation of 1.

This process was then iterated for subsequent generations up to 20. Data on the song entropy values of all individuals were saved in order to calculate numerically the change in mean song entropy from generation to generation (Δz), the exact covariance for the ancestors between their song entropy and their fitness ($cov(w, z)$), and the fitness-weighted average of the difference between an ancestor and their descendants ($E_w(\Delta z)$). In all cases, I verified that $5\Delta z = cov(w, z) + E_w(\Delta z)$, as expected.

Differences between the three scenarios.

In the first scenario (column a of figure 2), sampling of ancestors for each descendant was negatively weighted by song entropy, using R's sample() function. In the second scenario (column b), sampling was unweighted. In the third (column c), sampling was positively weighted.

The means of the distribution of transmission terms were 0 (column A); -0.75 (column B); and -2 (column C). These values were chosen numerically to give similar patterns of change over time to one another when coupled with negative, no, or positive selection respectively.

R code for these simulations is available at: https://osf.io/n695v/
